# Socioeconomic and ecological drivers of snakebite incidence in Mexico: A spatial analysis of risk factors

**DOI:** 10.1371/journal.pntd.0013582

**Published:** 2025-10-10

**Authors:** Rodrigo Rangel-Camacho, Carlos Yáñez-Arenas, J. P. Chippaux, Gerardo Martín

**Affiliations:** 1 Laboratorio de Ecología Geográfica - Unidad de Biología de la Conservación, Unidad Académica Sisal - Facultad de Ciencias, Universidad Nacional Autónoma de México, Parque Científico Chuburná, Yucatán, México; 2 Posgrado en Ciencias Biológicas, Universidad Nacional Autónoma de México, Ciudad de México, México; 3 Paris Cité University, Research Institute for Development, MERIT, Paris, France; 4 Departamento de Sistemas y Procesos Naturales, Escuela Nacional de Estudios Superiores Unidad Mérida, Universidad Nacional Autónoma de México, Mérida, México; Heidelberg University Medical Faculty Mannheim: Universitat Heidelberg Medizinische Fakultat Mannheim, GERMANY

## Abstract

**Background:**

Snakebite envenoming constitutes a significant public health challenge in tropical and subtropical regions, with Mexico reporting substantial incidence rates in the Americas. While previous investigations have documented the socioeconomic burden of snakebites, particularly in economically marginalized regions, a comprehensive understanding of the relative contributions of biological and socioeconomic determinants to spatial heterogeneity in snakebite incidence remains poorly understood. This study aimed to identify and quantify the main determinants of snakebite spatial heterogeneity across Mexico while accounting for potential reporting biases in surveillance data.

**Methods/principal findings:**

We implemented a rigorous Bayesian analytical framework utilizing a conditional autoregressive zero-inflated Poisson model to examine snakebite incidence across 2,463 Mexican municipalities. Our methodological approach integrated three critical components: environmental suitability indices for venomous snake species derived from refined species distribution models, socioeconomic vulnerability metrics, and healthcare accessibility parameters.

Social lag index (β = 0.308, 95% CI: 0.106-0.522), road network density (β = 0.376, 95% CI: 0.215-0.539), and environmental suitability for *Bothrops asper* (β = 0.268, 95% CI: 0.047-0.504) emerged as the primary factors explaining spatial variation in snakebite incidence. Healthcare facility distribution (β = 0.225, 95% CI: 0.126-0.326) was identified as a significant source of reporting bias. After controlling for this bias, our model revealed substantially different spatial pattern of risk, with elevated predicted incidence in urban centers and specific coastal regions not previously identified as high-risk areas.

**Conclusions:**

Our findings demonstrate that snakebite risk in Mexico is driven by a complex interaction between social vulnerability, infrastructure development, and the distribution of key venomous snake species. The identification of systematic reporting biases offers critical insights for optimizing surveillance protocols and implementing targeted interventions in high-risk municipalities.

## Author summary

Snakebites represent a significant health challenge in tropical and subtropical regions, including Mexico. Despite their importance, it remains poorly understood why some areas experience more snakebites than others. Our study aimed to identify the key factors that influence where snakebites occur across Mexico. We examined data from over 2,400 municipalities, considering environmental factors (like where venomous snakes live), social conditions (like poverty levels), and infrastructure (like road networks and hospitals). We found that three main factors explain most of the pattern: the level of social marginalization in a community, the density of road networks, and the environmental suitability for a particular venomous snake species called *Bothrops asper*. Importantly, we discovered that the official records of snakebite cases are strongly influenced by the availability of healthcare facilities - areas with more hospitals report more cases, which doesn’t necessarily mean they have more snakebites. When we accounted for this reporting bias, we identified different high-risk areas than those suggested by official reports. These findings can help authorities better target prevention efforts and distribute antivenoms to the communities that need them most.

## Introduction

Snakebite envenoming is a major public health burden with disproportionate impacts on rural populations in tropical and subtropical regions. Global estimates indicate approximately 1.8-2.7 million envenomings annually, resulting in 81,000–138,000 fatalities and 400,000 permanent disabilities [1]. Recognition of this burden prompted the World Health Organization to designate snakebite envenoming as a ‘neglected tropical disease’ in 2017, underscoring its relevance as a predominantly rural health concern with complex socioeconomic dimensions [2]. Within the Americas, Mexico exhibits particularly high incidence, with official surveillance systems documenting approximately 4,000 cases annually, while epidemiological models suggest actual incidence may reach 28,000 cases [3]. Beyond mortality, victims of snakebite frequently experience severe clinical sequelae, including tissue necrosis, neurotoxicity, coagulopathy, nephrotoxicity, and potential limb amputation [4,5]. These sequelae can also result in significant psychological trauma and economic hardship, which can perpetuate poverty cycles in affected populations [6,7].

Research on snakebite epidemiology has evolved along complementary trajectories, with distinct emphases on biological determinants and socioeconomic factors. Species distribution modeling approaches have demonstrated utility in characterizing environmental suitability for medically important snake species and associated bite risk in diverse geographic contexts including Ecuador [8], Iran [9], and Mozambique [10]. Concurrently, investigations in Brazil and other Latin American countries have identified critical socioeconomic risk factors, including urbanization gradients, agricultural occupation, housing quality, and marginalization indices. Within Mexico, correlative analyses in the state of Veracruz have documented significant associations between human marginalization indices, environmental suitability for venomous species (*Bothrops asper* and *Crotalus simus*), and spatial patterns of snakebite incidence [11]. Complementary epidemiological assessments have identified regional variation in medically significant species across states, with *C. molossus*, *C. atrox*, *C. durissus*, *Agkistrodon bilineatus*, and *B. asper* contributing substantially to snakebite burden in Oaxaca, San Luis Potosí, Hidalgo, and Puebla, respectively [12,13].

Despite this research efforts in Mexico, a significant methodological gap persists in quantifying the relative contributions and interactions between biological and socioeconomic determinants in explaining spatial heterogeneity of snakebite incidence. Previous investigations have frequently relied on descriptive epidemiology or correlative analyses that fail to account for spatial autocorrelation and reporting biases inherent in public health surveillance data. This limitation is particularly problematic given documented disparities between official reporting systems and community-based surveys in multiple countries [14,15], suggesting systematic underestimation of actual incidence. Furthermore, while both environmental and socioeconomic factors have independently demonstrated predictive value, the complex interplay between environmental suitability, human vulnerability factors, and healthcare accessibility remains inadequately characterized, limiting development of spatially explicit intervention strategies.

The methodological limitations in quantifying risk factors demand the development of more sophisticated analytical approaches, particularly in the context of Mexico’s epidemiological surveillance system, where systematic underreporting and data inconsistencies present substantial challenges. The marked discrepancy between officially documented cases and model-based estimates [3,16] highlights the inadequacy of conventional analytical methods that fail to account for reporting biases. Furthermore, the inherent spatial heterogeneity of snakebite incidence, influenced by region-specific environmental conditions and socioeconomic gradients, requires spatially explicit modeling frameworks that can disentangle true risk patterns from artifacts of surveillance coverage. Beyond identifying spatial patterns, however, elucidating the specific causal mechanisms that drive human-snake encounters and subsequent envenomation events is essential for developing effective preventive strategies. Understanding how environmental suitability for venomous species interacts with human vulnerability factors to produce observable snakebite patterns represents a critical knowledge gap with significant practical implications for antivenom distribution, healthcare resource allocation, and implementation of targeted preventive interventions [17–19].

We hypothesize that spatial heterogeneity in snakebite incidence across Mexican municipalities is driven by the interaction between environmental and socioeconomic factors through two main mechanisms: First, environmental suitability determines snakes abundance and thus the probability of human-snake encounters, varying species of relevance in bite occurrence depending on the region of the country; and second social lag influences human exposure to snakes through occupational activities and limited access to preventive healthcare in marginalized areas. From this hypothesis, we derive the following predictions: 1) Municipalities with higher environmental suitability for *B. asper* will show higher snakebite incidence, 2) The relationship between social lag and snakebite incidence will be positive but non-linear, reaching a maximum at intermediate levels of marginalization where human activities overlap with snake habitats, 3) The effect of environmental suitability on snakebite incidence will be strongest in municipalities with high social lag, and 4) When controlling for hospital availability bias, the spatial pattern of snakebite risk will more closely reflect the interaction between environmental suitability and social lag than the raw incidence data.

To test these hypotheses and their derived predictions, we implemented a Bayesian spatial modeling framework incorporating environmental suitability indices for venomous snake species derived from refined species distribution models, socioeconomic vulnerability metrics, and healthcare accessibility parameters. This approach explicitly accounts for spatial autocorrelation while addressing zero-inflation patterns characteristic of rare-event reporting in surveillance systems. Through this methodologically rigorous analysis, we aim to characterize the multifactorial determinants of snakebite risk in Mexico and provide an empirical foundation for evidence-based public health interventions in high-risk regions.

## Methodology

### Data collection

We obtained the total number of snakebite (SB) cases in 2018 for all municipalities in the 32 states of Mexico through formal request for public information to the Ministry of Health. We selected 2018 as our analysis year based on the comprehensive availability of both epidemiological and socioeconomic data for this period, allowing for optimal integration of multiple data sources. We calculated population-adjusted snakebite incidence (SBI) rates by dividing the total number of reported snakebites by the corresponding municipal population count and multiplying by 100,000 [20]. We acquired population data from WorldPop [21] to address geographical inconsistencies observed in national government censuses, particularly in selected Oaxacan municipalities where official census data yielded logically impossible incidence calculations (reported snakebite cases exceeding total population counts).

### Socioeconomic and infrastructure factors

We quantified socioeconomic vulnerability using the Social Lag Index (SLI) at the municipal level, obtained from the National Institute of Statistics, Geography and Informatics (INEGI) 2020 Population and Housing Census [22]. We operationalized healthcare accessibility through georeferenced databases of public hospitals and clinics (“IMSS” and “Salud y Bienestar”) distribution per municipality. We represented human penetration into potential snake habitats through comprehensive road network data from 2018, obtained from CentroGeo CONAHCYT 2020 database (https://idegeo.centrogeo.org.mx/interactive/layers). Both healthcare facilities and transportation networks were available as vector data in ESRI shapefile format. For analytical purposes, we extracted precise counts of healthcare facilities and number of road segments for each of the 2,463 municipalities across Mexico.

### Environmental suitability for medically important snake species

We assessed environmental suitability for venomous snake species using species distribution models (SDMs) [23,24] of 38 medically relevant vipers from the genera Agkistrodon, Bothrops, and Crotalus known to occur within Mexican territory. We obtained these models from the VenomMaps repository (https://rhettrautsaw.app/shiny/VenomMaps/) [25], which provides standardized and validated distribution models specifically developed for venomous snake species. We aggregated environmental suitability values (1 km spatial resolution) to municipal boundaries using the R package terra [26]. For each municipality, we calculated multiple distribution metrics (mean, coefficient of variation, median, and maximum) to capture different dimensions of potential species presence. We selected median suitability values for final analyses as they provide the most robust central tendency measure resistant to outlier values that could bias results. We included only municipalities with at least one grid cell with non-zero suitability values in species-specific analyses.

### Data analysis

#### Exploratory analysis and variable selection.

We evaluated initial variable relationships using Spearman’s rank correlation coefficients to assess associations between reported SB incidence and potential predictor variables (snake species suitability and socioeconomic factors). We selected this non-parametric approach to accommodate potential non-linear relationships and non-normal distributions in ecological and socioeconomic data [27].

For snake species selection, we implemented a two-stage filtering process. First, we identified species with statistically significant correlations (p < 0.05) with snakebite incidence. Second, we applied a minimum correlation threshold of ρ ≥ 0.1, because weak correlations tend to have biological relevance in complex ecological systems [28,29]. This threshold represents a balance between statistical significance and ecological meaningfulness, as demonstrated in previous snakebite epidemiology studies [8,30].

We systematically assessed multicollinearity among predictor variables using a multi-faceted approach. We initially computed pairwise Spearman correlation coefficients between all predictors, identifying highly correlated variable pairs (ρ > 0.7) for further examination. This threshold aligns with established guidelines in ecological modeling [31,32]. For variable pairs exceeding this correlation threshold, we retained the variable demonstrating stronger univariate relationship with snakebite incidence based on both statistical strength and theoretical relevance. Additionally, we calculated Variance Inflation Factors (VIFs) for all variables in preliminary models, with VIF > 5 considered indicative of severe multicollinearity requiring variable elimination. This rigorous variable selection process resulted in 36 variables being excluded from consideration in Poisson model components and 37 variables from zero-inflation components. We provide two examples of correlation results and scatterplots in S1 and S2 Figs and table with VIF results of predictive variables employed in the model S3 Fig.

#### Model development and selection.

Prior to model fitting, we examined the functional form of relationships between each predictor and snakebite incidence using scatter plots with locally estimated scatterplot smoothing (LOESS) to evaluate linearity assumptions. Based on these visualizations and theoretical considerations, we applied appropriate transformations to predictor variables, including logarithmic transformation for population density and quadratic terms for roads and SLI due to evident non-linear relationships (S1 and S2 Figs). We subsequently standardized all variables (mean = 0, SD = 1) to facilitate comparison of effect sizes.

We systematically generated and evaluated a comprehensive set of candidate models representing all possible combinations of non-collinear variables, including polynomial terms up to third-order where biologically justified. We guided the inclusion of polynomial terms by both visualization of generalized linear models (GLMs) and theoretical understanding of potential non-linear ecological relationships. We employed orthogonal polynomials to address potential collinearity issues among polynomial terms. We retained higher-order polynomials (third-order) only when they significantly improved model fit (ΔAIC > 4) and demonstrated biologically interpretable patterns.

For initial model screening, we fit negative binomial generalized linear models (GLMs) to snakebite count data using log-transformed human population as an offset term [27,33], implemented through the R package MASS [34]. We specifically considered zero-inflated models due to the high prevalence of municipalities with no reported snakebite cases (72.4% of observations), which could represent either true absence of snakebites or reporting failures [35].

We followed a systematic approach to model selection, incorporating both statistical criteria and theoretical considerations. We initially identified the 10 models with lowest AIC values, then evaluated each formula individually based on: 1) statistical significance of estimated parameters, 2) overall model performance metrics, and 3) biological plausibility of included variables and their interactions. We gave priority to models with significant positive estimated effects in the Poisson component, ensuring that at least one snake species median suitability was retained in the final model to address our specific research questions regarding the contribution of environmental suitability to snakebite risk.

To identify factors potentially influencing the zero-generating process, we performed parallel binomial GLM analyses on binarized snakebite data (presence/absence). We followed similar criteria for model selection for the zero-inflation component, prioritizing models with all effects significantly different from zero.

#### Bayesian spatially explicit incidence model.

Following rigorous variable selection, we implemented a Bayesian conditional autoregressive (CAR) model [36] with zero-inflated Poisson error distribution using the R package CARBayes [37]. We specifically selected this modeling framework to address three critical methodological challenges: 1) spatial autocorrelation in disease incidence patterns, 2) excess zeros characteristic of rare health events, and 3) the need to quantify uncertainty in parameter estimates. The model structure follows a conditional autoregressive specification for spatial random effects:


ψk=ϕk+θk

$$\varphi k|\varphi -k,W,\tau^{\text{2}}\sim N\left(\frac{\sum_{i=\text{1}}^{k}wki\varphi i}{\sum_{i=\text{1}}^{k}wki},\frac{\tau^{\text{2}}}{\sum_{i=\text{1}}^{k}wki}\right)$$

$$\theta k\sim N(\text{0}{,\sigma }^{\text{2}})$$

$$\tau^{\text{2}},\sigma^{\text{2}}\sim Inverse-Gamma(\text{1},\text{0}.\text{0}\text{0}\text{1})$$

Where ϕk represents the total spatial random effect for municipality k, decomposed into a spatially structured component (ϕk) and an unstructured component (θk). The spatially structured component follows a conditional autoregressive distribution dependent on neighboring values, weighted by the spatial neighborhood matrix W. The parameters $\tau^{\text{2}}$ and $\sigma^{\text{2}}$ represent the variance components for structured and unstructured spatial variation, respectively, with Inverse-Gamma prior distributions.

The zero-inflated Poisson distribution for the response variable is specified as:


$$\text{Pr}(Y=\text{0})=\pi_{i}+\left(\text{1}-\pi_{i}\right)e^{-\lambda i}$$

$$\text{Pr}\left(Y=y_{i}>\text{0}\right)=(\text{1}-\pi )\frac{\lambda^{y_{i}}e^{-\lambda_{i}}}{yi!}$$

Where $\pi_{i}$ represents the probability of structural zeros (municipalities with no risk of snakebites) and $\lambda_{i}$ is the Poisson mean for municipality i. We defined the spatial neighborhood structure using first-order queen contiguity (municipalities sharing at least one boundary point were considered neighbors), with row-standardized weights.

Following preliminary model testing, we refined the model specification by removing certain quadratic terms that showed convergence issues. We assessed model fit using the Deviance Information Criterion (DIC), posterior predictive checks, and residual diagnostics. We evaluated convergence through examination of trace plots, effective sample sizes, and potential scale reduction factors (PSRF).

#### Model validation and prediction.

Our model validation strategy incorporated multiple complementary approaches. We compared posterior predictions with observed snakebite data using: 1) a linear regression model between observed and predicted values; 2) the Kling-Gupta efficiency index (KGE) [32], which provides a comprehensive assessment of correlation, bias, and variability ratio; and 3) spatial autocorrelation analysis of model residuals using Moran’s I [38].

For identifying maxima in the relationships between predictor variables and snakebite incidence, we employed partial derivatives of the incidence function with respect to SLI and road density. Given a function:


$$f(x)=\beta_{i}x+\beta_{j}x^{\text{2}}+C$$

Where x represents SLI or road density and β represents the regression coefficients, we calculated:


$$f´(x)=\beta_{i}+\text{2}\beta_{j}x$$

Setting f´(x) = 0 and solving for x, we determined the value at which the function reaches its maximum: $x^{\prime }=\frac{\beta_{i}}{\text{2}\beta_{j}}$

To approximate the spatial variation of snakebite risk while controlling for potential healthcare accessibility bias, we generated model predictions with the number of hospitals per municipality set to zero across all locations, maintaining all other parameters at their observed values, using the general functional form:


$$\text{log}(y)=\beta_{\text{0}}+\beta_{\text{1}}x_{\text{1}}+\beta_{\text{2}}x_{\text{2}}+\ldots +\beta_{i}x_{i}+\rho +\text{log}(pop)$$

Where ρ represents the autoregressive spatial random effect. These adjusted predictions provide insight into underlying risk patterns independent of healthcare reporting bias, allowing more accurate identification of high-risk areas that may be underrepresented in official statistics due to limited healthcare infrastructure.

## Results

### Descriptive analysis of snakebite distribution in Mexico

**Fig 1:**
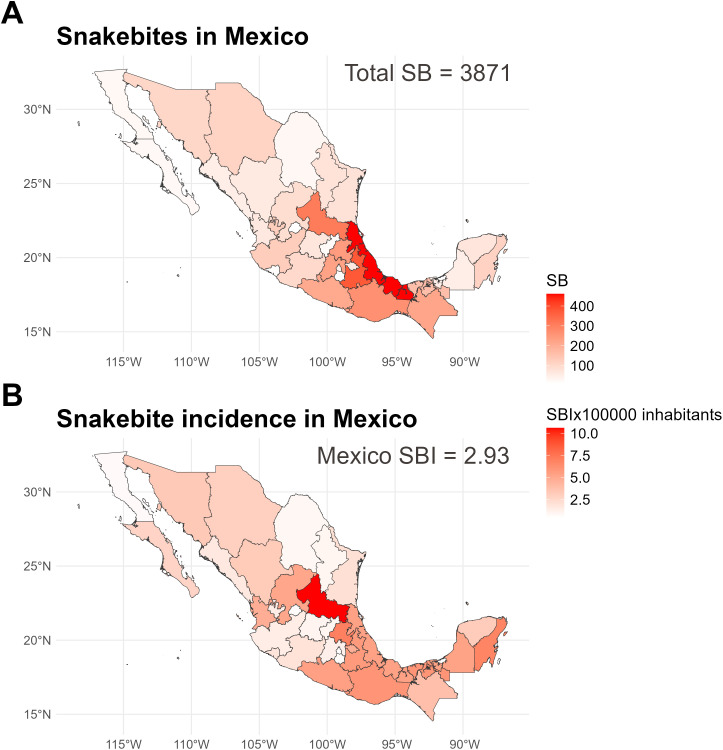
Geographic distribution of snakebite cases in Mexico (2018). (A) Total number of reported snakebites per state. (B) Population-adjusted snakebite incidence per 100,000 inhabitants. Colors indicate intensity of occurrence, with darker shades representing higher values. The base layer of the map showing Mexican municipalities was obtained from: https://idegeo.centrogeo.org.mx/geovisor. (INEGI, 2024). Terms of use: *https://www.inegi.org.mx/contenidos/inegi/doc/terminos_info.pdf*.

In 2018, a total of 3,737 snakebite cases were reported across Mexico. Aggregated by state, the highest number of snakebites was reported in Veracruz (461 cases), followed by Oaxaca (353) and San Luis Potosí (301). However, when adjusted for population size, San Luis Potosí had the highest incidence of 10.61 cases per 100,000 inhabitants, followed by Hidalgo (6.97) and Quintana Roo (6.75) (Fig 1A and 1B).

At the municipal level, 680 of 2,463 municipalities (27.6%) reported at least one snakebite case, with substantial geographical heterogeneity in reporting patterns. The municipalities with the highest absolute number of reported cases were Centro in Tabasco (65 cases), Xalapa in Veracruz [44], and San Luis Potosí in San Luis Potosí [42].

### Exploratory correlations with potential drivers

Municipal snakebite incidence showed significant positive correlations with both hospital density (ρ = 0.379, P < 2.2 × 10^-16^) and road network coverage (ρ = 0.202, P < 2.2 × 10^-16^). Among the 38 snake species analyzed, only three showed positive correlations with incidence (ρ ≥ 0.1): 1) *Crotalus ehecatl*, present in 209 municipalities (ρ = 0.256, P < 1 × 10^-3^); 2) *Bothrops asper*, present in 1,012 municipalities (ρ = 0.217, P < 2.4 × 10^-12^); and 3) *Crotalus tlaloci*, present in 291 municipalities (ρ = 0.129, P = 0.027) (S1 and S2 Figs as examples).

### Model parameters and estimates

From an initial set of 57 candidate models, only one formula met all predefined inclusion criteria:


$$\text{SB2018}\;\sim \;\text{roads}\;+\;{\text{roads}}^{\text{2}}\;+\;\text{hospitals}\;+\;\text{SLI}\;+\;{\text{SLI}}^{\text{2}}\;+\;\text{B}.asper\text{ES}\;+\;\text{offset}(\text{log}(\text{pop2018}))$$

Zero probability equation (Ω) ~ SLI^2 ^+ *B. asper* ES^2^

(see S4 Fig for detailed model diagnostics).

Road density showed the strongest positive association with snakebite occurrence (β = 0.376, 95% CI: 0.215-0.539), followed by social lag index (β = 0.308, 95% CI: 0.106-0.522), and hospital availability (β = 0.225, 95% CI: 0.126-0.326). Environmental suitability for *B. asper* also showed a significant positive association (β = 0.268, 95% CI: 0.047-0.504). Both road density and social lag index exhibited significant negative quadratic terms (roads^2^: β = -0.027, 95% CI: -0.043 to -0.012; SLI^2^: β = -0.123, 95% CI: -0.207 to -0.041) (Table 1).

**Table 1:** Parameter estimates from the zero-inflated Poisson conditional autoregressive model of snakebite occurrence in Mexico (2018). Estimates include posterior means and 95% credible intervals (2.5% and 97.5% quantiles) for fixed effects, zero-inflation parameters (Ω), and variance components (τ² for spatial variation, σ² for nonspatial variation). Environmental suitability (ES) was calculated for *B. asper*. SLI: Social Lag Index.

	Mean	2.5%	97.5%	n.effective	PSRF (upper 95% CI)
Intercept	-11.404	-11.559	-11.2602	2513.1	1
Roads	0.376	0.215	0.539	1487.1	1
roads^2^	-0.027	-0.043	-0.012	1865	1
hospitals	0.225	0.126	0.326	2832.1	1
SLI	0.308	0.106	0.522	1480.1	1
SLI^2^	-0.123	-0.207	-0.041	2155.1	1
*B. asper* ES	0.268	0.047	0.504	3127.5	1
Ω Intercept	-251.63	-654.808	-14.257	3457.9	1
Ω SLI^2^	-4.415	-23.507	16.451	3593.6	1
Ω *B. asper* ES^2^	-4.538	-24.113	17.44	3491.8	1
Τ^2^	2.076	1.114	3.244	2362.3	1
σ^2^	1.907	1.542	2.279	2875.0	1

The partial derivative analysis revealed maximum snakebite numbers at values of 665.5 roads per municipality and -0.246 SLI units.

### Model performance metrics

The linear regression between predicted and observed snakebite cases yielded an R² value of 0.99. The model correctly classified snakebite occurrence (presence/absence) in 2,073 out of 2,463 municipalities (84.09%), with a prediction error within ± 0.53 cases in municipalities where snakebites occurred (S5-S7 Figs).

For population-adjusted incidence rates per 100,000 inhabitants, the mean difference between reported and predicted values was 0.12 (S7 Fig). The Kling-Gupta efficiency index (KGE) was 0.967, and spatial autocorrelation analysis of model residuals yielded a Moran’s I value of 0.119.

**Fig 2:**
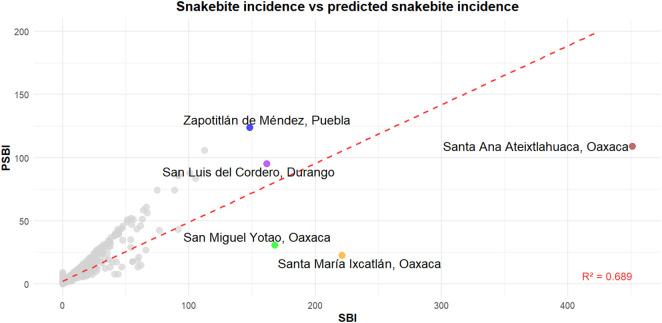
Model validation: observed (SBI) versus predicted snakebite incidence (PSBI) rates. The solid line represents the 1:1 relationship. Labeled points indicate municipalities with substantial deviation from model predictions: Zapotitlán de Méndez, Puebla (blue), San Luis del Cordero, Durango (purple), San Miguel Yotao, Oaxaca (green), Santa María Ixcatlán, Oaxaca (yellow) and Santa Ana Ateixtlahuaca, Oaxaca (brown).

The model substantially underestimated incidence in five municipalities with exceptionally high observed values: Zapotitlán de Méndez (Puebla), San Luis del Cordero (Durango), San Miguel Yotao (Oaxaca), Santa María Ixcatlán (Oaxaca), and Santa Ana Ateixtlahuaca (Oaxaca) (Fig 2).

### Hospital bias-controlled predictions

**Fig 3:**
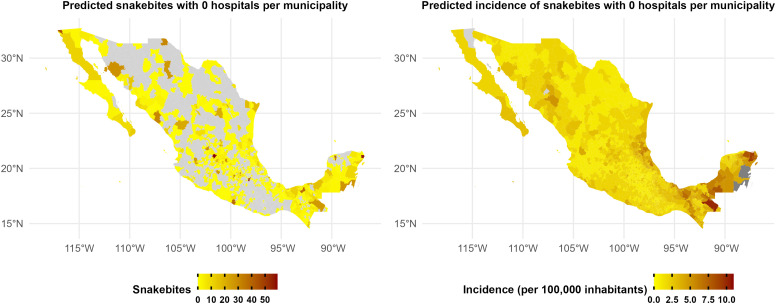
Spatial distribution of predicted snakebite risk after controlling for hospital-related reporting bias. This map shows model predictions when hospital density is standardized to zero across all municipalities while maintaining spatial autocorrelation effects, thereby revealing the underlying risk pattern independent of healthcare access. Grey represents municipalities with zero counts of predicted snakebites or predicted snakebite incidence. The base layer of the map showing Mexican municipalities was obtained from: https://idegeo.centrogeo.org.mx/geovisor. (INEGI, 2024). Terms of use: *https://www.inegi.org.mx/contenidos/inegi/doc/terminos_info.pdf*.

After standardizing hospital density to zero across all municipalities, most previously identified high-risk municipalities showed reduced risk estimates, with the exception of Centro (Tabasco). The municipalities with the highest predicted risk after controlling for hospital-related reporting bias were León (Guanajuato), Benito Juárez (Quintana Roo), Puebla (Puebla), Tijuana (Baja California), and Centro (Tabasco) (Fig 3).

## Discussion

Our findings reveal a complex interplay of socioeconomic and environmental factors driving snakebite incidence in Mexico. The significant influence of social lag index, road density, and *B. asper* habitat suitability demonstrates that snakebite risk emerges from the intersection of human vulnerability, infrastructure development, and ecological conditions. Particularly noteworthy is how hospital density distorts our understanding of the spatial distribution of snakebite burden, creating systematic reporting biases that mask the true epidemiological pattern.

These findings partially support our initial hypothesis. As predicted, environmental suitability of venomous snakes combined with sociodemographic conditions explains most of the observed variability in snakebite burden across Mexico. We correctly anticipated that only a small subset of snake species would drive spatial patterns in snakebite incidence. However, we found that a single species *B. asper* was sufficient to explain nationwide risk variability, which represents an even stronger pattern of species dominance than we initially hypothesized. This finding aligns with previous research in Latin America [39], where snakebite incidence patterns are typically driven by a few abundant, widely distributed species rather than by diverse assemblages of venomous snakes. *B. asper* emerges as the species that best explains snakebites in Mexico, likely due to its adaptative capability to human-modified areas, including regions with certain levels of disturbance along the agricultural frontier, making its presence in or near human dwellings not uncommon [40]. Furthermore, this species constantly moves in search of food and is difficult to spot, as it selects understory or humid areas for hunting [41,42]. Coupled with its irritable disposition [42] can lead to negative interactions between humans and this species.

The geographic distribution of *B. asper* poses an important constraint on our model interpretation (S8 Fig). While this species effectively explains snakebite patterns in southern Mexico where it occurs, its absence from northern and central regions creates an analytical challenge. Interestingly, species such as *Crotalus atrox* and *C. molossus*, despite their wide distribution, did not show significant correlations with snakebite incidence (ρ = 0.02, P = 0.45; and ρ = -0.12, P = 0.00, respectively). In these *B. asper*-absent regions, our model still predicted snakebite patterns with reasonable accuracy despite lacking species-specific information, suggesting that socioeconomic factors may compensate for the explanatory power of species distribution data. This regional variation in explanatory factors highlights the context-dependency of snakebite risk and suggests a potential north-south gradient in risk determinants: ecological factors may predominate in species-rich southern regions, while socioeconomic vulnerability and exposure patterns may be more influential in northern areas where fewer medically relevant snake species occur.

Beyond snake species distribution, our results demonstrate that infrastructure and social factors are the primary drivers of snakebite variability in Mexico. The standardized coefficients reveal a clear hierarchy of influence: road density (β = 0.376) exerts the strongest effect, followed by social lag index (β = 0.308) and hospital availability (β = 0.225). This finding underscores that snakebite incidence is fundamentally a socio-ecological phenomenon, with human-built environments and social vulnerability creating the conditions for human-snake encounters. The prominent role of road networks is particularly noteworthy but requires careful interpretation. As previous studies have suggested [17,18], roads have dual and potentially contradictory effects—they may simultaneously increase human penetration into snake habitats (increasing exposure risk), improve access to healthcare facilities (increasing reporting rates), an increase in terrestrial transport routes within agricultural areas has been observed to correlate with increased agricultural production [43,44]. In turn, agricultural production is frequently associated with snakebites, as snakes and humans often share the same time and space in these environments [45,46]. Habitat degradation may reduce snake populations in some areas; however, in other contexts, it can displace snakes into human settlements or agricultural zones, potentially increasing the risk of snakebites.

The non-linear relationships we identified between snakebite incidence and both road density and social lag index reveal important threshold effects in human-snake conflict. The peak snakebite risk at intermediate levels of road density (665.5 roads per municipality) likely represents an optimal balance where human access to snake habitats is maximized while natural vegetation remains sufficiently intact to support snake populations. Similarly, the peak at moderate social lag values (-0.246 SLI units) suggests that the most vulnerable populations are not those in extreme poverty (who may have limited healthcare access and thus underreport bites) or affluent communities (with minimal exposure to snake habitats), but rather those in transitional socioeconomic conditions with both significant environmental exposure and sufficient access to report cases.

As snakebite is classified as a neglected tropical disease, we observed the expected socioeconomic pattern typical of this disease category: a higher snakebite burden in populations with high social lag index. This finding is consistent with previous observations in Veracruz [11] regarding the relationship between snakebite incidence and human marginalization. Our results extend these local observations to a national scale, confirming that the socioeconomic dimension of snakebite risk is consistent across diverse geographical contexts.

Hospital availability emerged as a crucial factor in our analysis, but primarily as a source of reporting bias rather than a causal determinant of snakebite risk. Annual snakebite case estimates for Mexico vary significantly across studies and reporting periods, ranging from 788 to 4,000 cases per year [16,47]. These disparities represent a significant methodological challenge, as they may not accurately reflect true epidemiological patterns. Short temporal periods of analysis are particularly problematic, as they often fail to capture the full range of variability in snakebite incidence across years. Interannual variability may result from changes in healthcare access and event reporting policies, personnel, or data infrastructure rather than actual changes in snakebite occurrence. However, variations in the incidence of snakebite related to climatic variations cannot be ruled out [48].

Residents of municipalities with limited healthcare access likely seek treatment in nearby municipalities with better hospital availability, creating a spatial mismatch between where bites occur and where they are reported. Nevertheless, by controlling for this reporting bias, we were able to infer the true underlying pattern of snakebite risk at the municipal level (Fig 3). When we standardized hospital density to zero across all municipalities, the spatial pattern of predicted snakebite risk changed substantially (Fig 3), revealing different high-risk areas compared to the raw data. In light of these inherent limitations in healthcare data records, there is a widespread need to adopt innovative methodological approaches, such as those demonstrated in this study, to identify municipalities with underreported or overreported snakebite cases.

Our approach enables the identification of municipalities with significant discrepancies between reported and predicted cases. For instance, we identified several municipalities with environmental and social conditions conducive to high snakebite risk that were previously overlooked in official reports, including León (Guanajuato), Benito Juárez (Quintana Roo), Puebla (Puebla), Tijuana (Baja California), and Centro (Tabasco). This finding has substantial implications for public health planning and resource allocation, suggesting that antivenom distribution and preventive interventions may need to be recalibrated to target these previously unrecognized high-risk areas.

We acknowledge several methodological limitations in our study. First, zero-inflated Poisson models tend to underestimate extreme values due to their short-tailed distribution [49,50] Additionally, the quadratic terms for social lag index (SLI2) and environmental suitability for B. asper (ES2) exhibit wide confidence intervals that cross zero, indicating uncertainty about their isolated statistical significance. Nonetheless, we retained these variables to ensure model convergence and due to the theoretical expectation that social and ecological gradients should influence both the occurrence and absence of snakebites across municipalities. Their inclusion allowed for a more robust assessment of underlying risk patterns, even in areas with few or no reported cases. In our analysis, the model substantially underestimated incidence in five municipalities with exceptionally high observed values (Fig 2), which highlights the challenge of accurately predicting localized hotspots of snakebite risk. Future researches could conduct meticulous epidemiological studies of those underestimated municipalities to identify localized risk factors, such as ecological conditions or specific human activities, to explore the potential factors that explain the variability of the CAR component, with a particular focus on approaching extreme values. Second, our analysis was limited to a single year (2018), which may not be fully representative of the spatial variability in snakebite patterns and many conditions and characteristics may have changed over the past seven years. Multi-year analyses would provide more robust insights into both consistent risk factors and temporal dynamics.

For future research encompassing longer time periods, we recommend incorporating additional socioecological variables such as land-use change, population growth patterns [19], regional economic activities [19,51,52], and not only the Social Lag Index (SLI) as a broad proxy for socioeconomic vulnerability. These factors may help explain local variations in snakebite risk that our current model could not fully capture. Additionally, we suggest that health authorities collect more detailed spatial information about snakebite incidents, recording not only the municipality where treatment was sought but also the specific locality where the bite occurred. This finer-scale spatial data would substantially improve our ability to model snakebite risk and identify causal mechanisms.

In conclusion, our study provides a comprehensive framework for understanding the complex socioecological determinants of snakebite risk in Mexico. By revealing the interplay between environmental suitability, social vulnerability, and infrastructure development, our findings contribute to shifting the focus of snakebite management from treatment to prevention [53]. The identification of systematic reporting biases and previously unrecognized high-risk areas offers valuable insights for optimizing resource allocation and developing targeted interventions. Our findings can contribute to improving snakebite management by informing the geographic prioritization of surveillance programs and antivenom distribution. High-incidence areas with limited healthcare infrastructure and high social lag should be targeted for increased surveillance, health education, and strategic placement of antivenom stocks to reduce morbidity and mortality. Ultimately, this approach can help mitigate both the health and economic consequences of snakebite envenoming through improved prevention strategies [54–56], environmental education, and enhanced healthcare access [57,58].

## Supporting information

S1 Fig Scatter plot with regression and smooth lines of with Spearman’s correlation coefficient between hospitals (logical_hm) and snake bite incidence. (TIF)

S2 Fig Scatter plot with regression and smooth lines of with Spearman’s correlation coefficient between *Crotalus ehecatl* (medCehe) and snake bite incidence. (TIF)

S3 Fig Table of explanatory variables used in the final model with their VIF values. (TIF)

S4 Fig The model diagnostics of the final model with residuals histogram (top), and scatter plot of residual vs fitted values (bottom). (TIF)

S5 Fig **Comparison between model predictions and observed snakebite data across Mexican municipalities (2018).** Total snakebite counts: model predictions (main map) and observed cases (inset). The base layer of the map showing Mexican municipalities was obtained from: https://idegeo.centrogeo.org.mx/geovisor. (INEGI, 2024). Terms of use: https://www.inegi.org.mx/contenidos/inegi/doc/terminos_info.pdf
(TIF)

S6 Fig **Comparison between model predictions and observed snakebite data across Mexican municipalities (2018).** Population-adjusted snakebite incidence (main map) and observed cases (inset). The base layer of the map showing Mexican municipalities was obtained from: https://idegeo.centrogeo.org.mx/geovisor. (INEGI, 2024). Terms of use: https://www.inegi.org.mx/contenidos/inegi/doc/terminos_info.pdf
(TIF)

S7 Fig **Map of municipalities with differences (red) between SB and PSB by the CAR model with a linear model between SB and PSB.** The base layer of the map showing Mexican municipalities was obtained from: https://idegeo.centrogeo.org.mx/geovisor. (INEGI, 2024) Terms of use: https://creativecommons.org/licenses/by-nc/2.5/mx/.
(TIF)

S8 Fig **Map of municipalities with *B. asper* suitability different of 0 in red.** The base layer of the map showing Mexican municipalities was obtained from: https://idegeo.centrogeo.org.mx/geovisor. (INEGI, 2024). Terms of use: https://www.inegi.org.mx/contenidos/inegi/doc/terminos_info.pdf
(TIF)

S1 Data Table with raw snakebite cases in Mexico (data_snkbtsmx.zip) (ZIP)
