# Supplementary figures and images for: Socioeconomic and ecological drivers of snakebite incidence in Mexico: A spatial analysis of risk factors
Source: PLoS Negl Trop Dis. 2025 Oct 10;19(10):e0013582. doi: 10.1371/journal.pntd.0013582 (PMC12513608; doi:10.1371/journal.pntd.0013582)

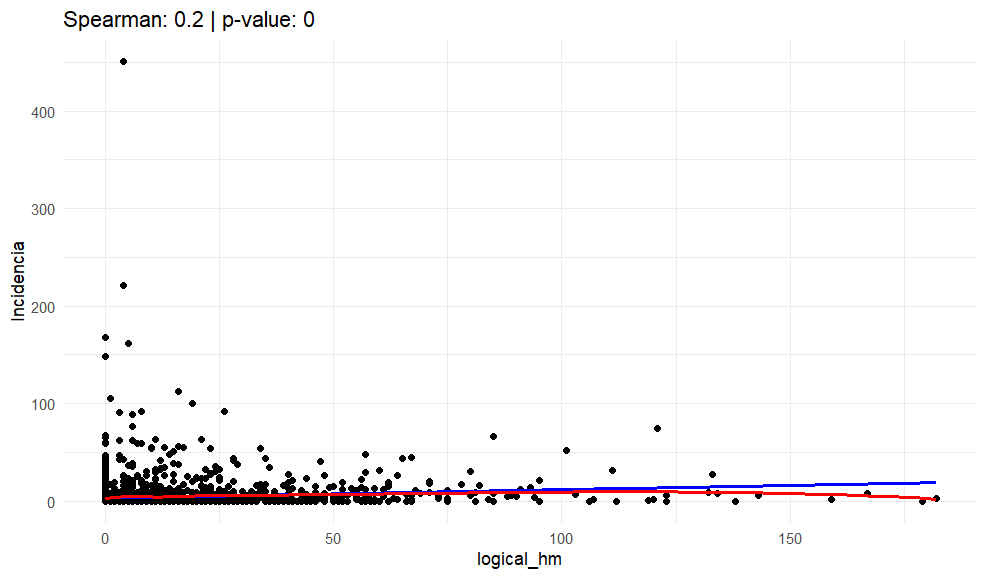

Supplement: S1 Fig — (TIF) [file pntd.0013582.s001.tif]

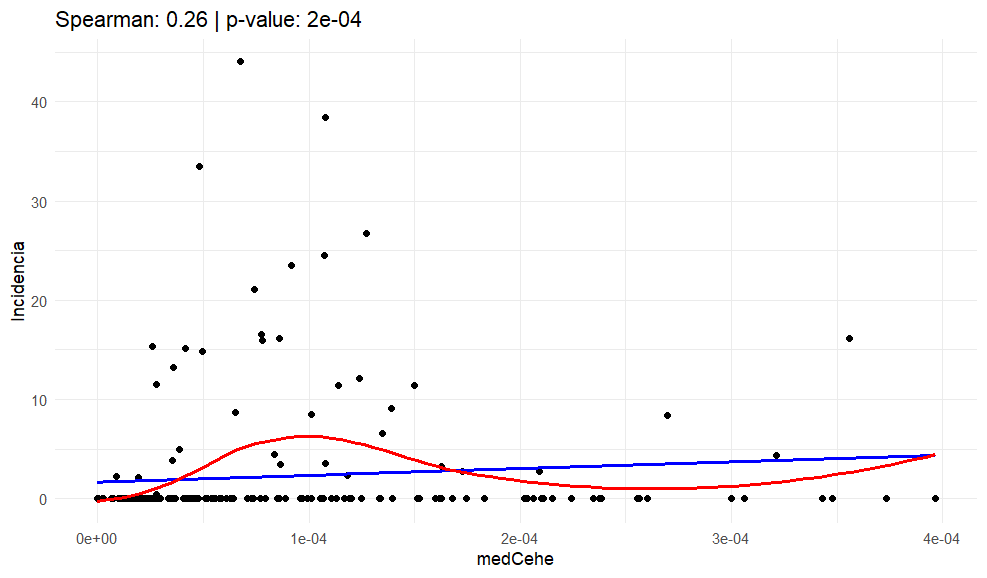

Supplement: S2 Fig — (TIF) [file pntd.0013582.s002.tif]

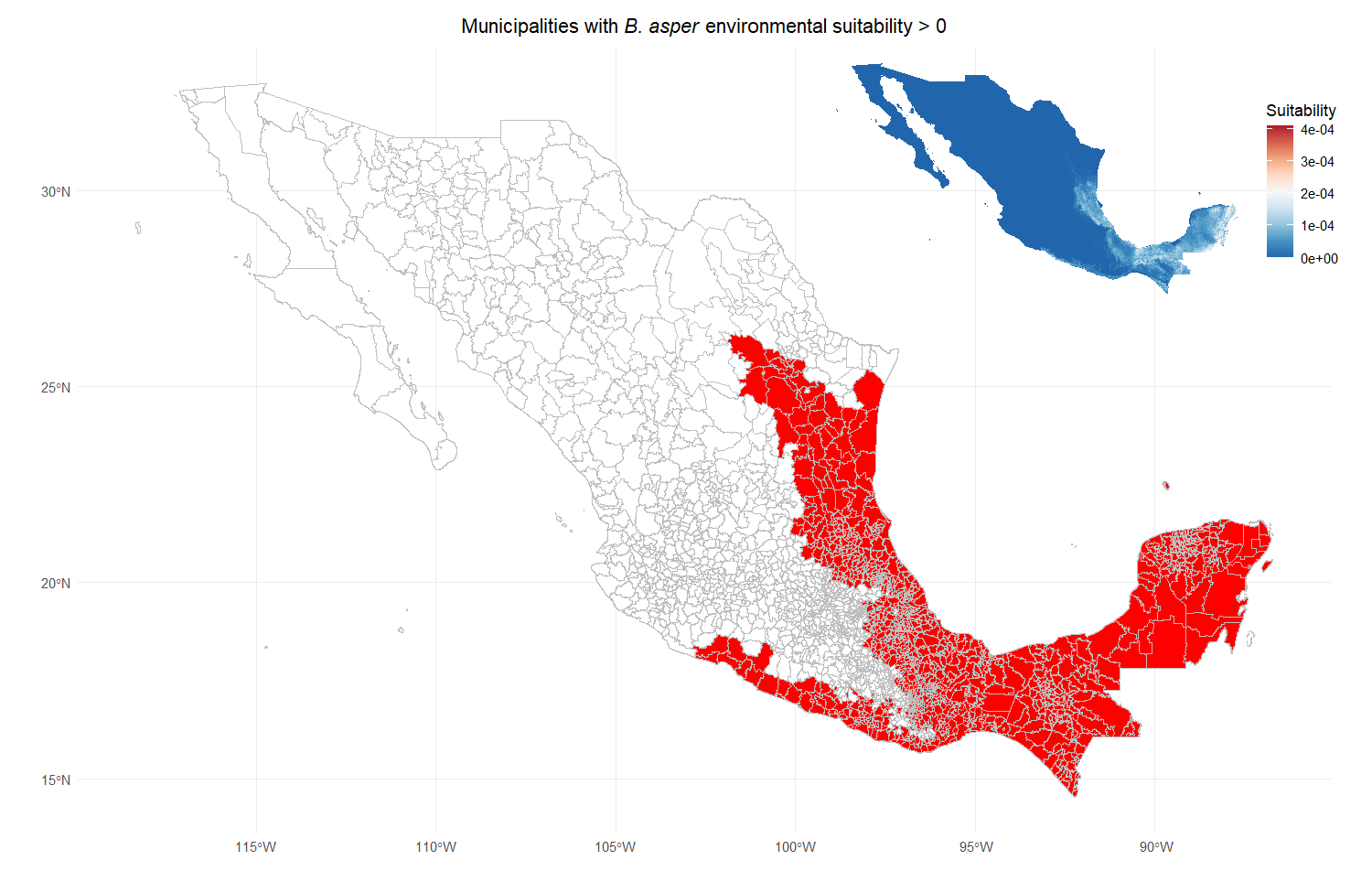

Supplement: S3 Fig — (TIF) [file pntd.0013582.s009.tif]

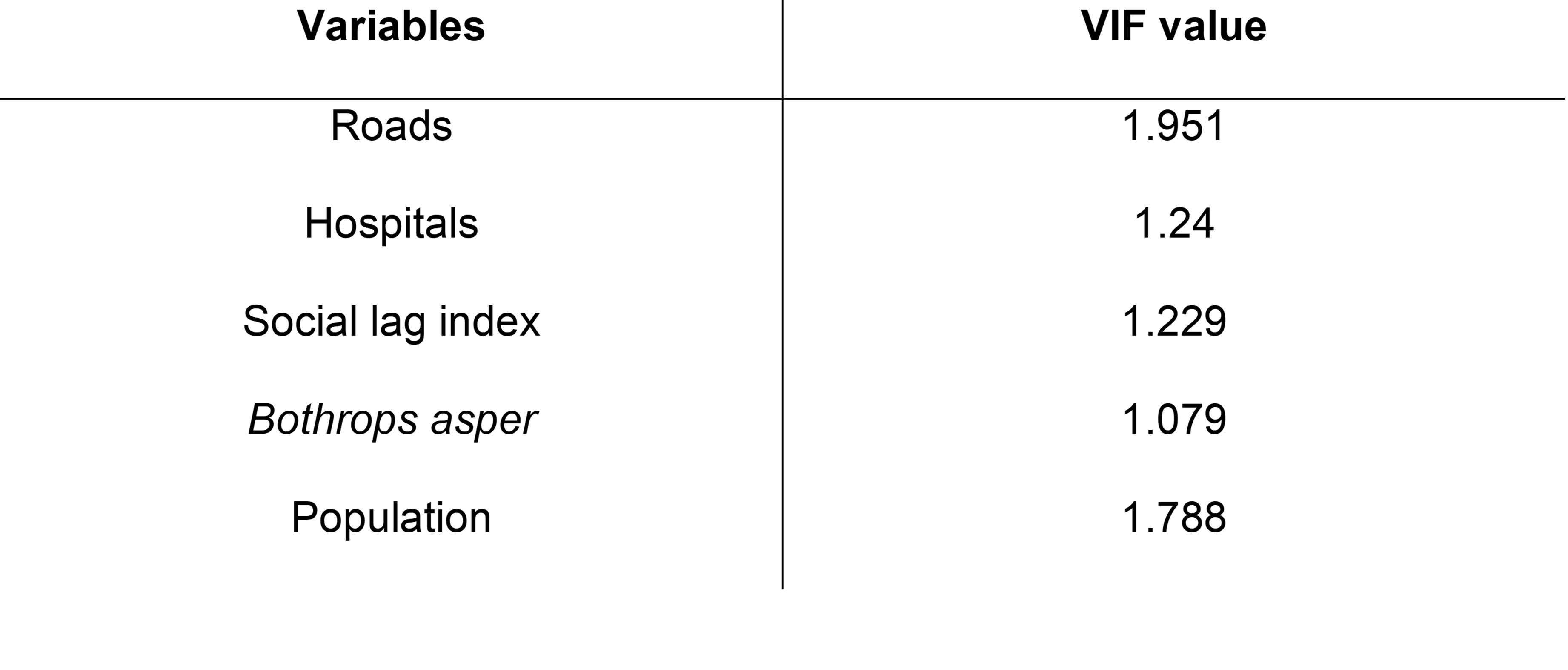

Supplement: S4 Fig — (TIF) [file pntd.0013582.s003.tif]

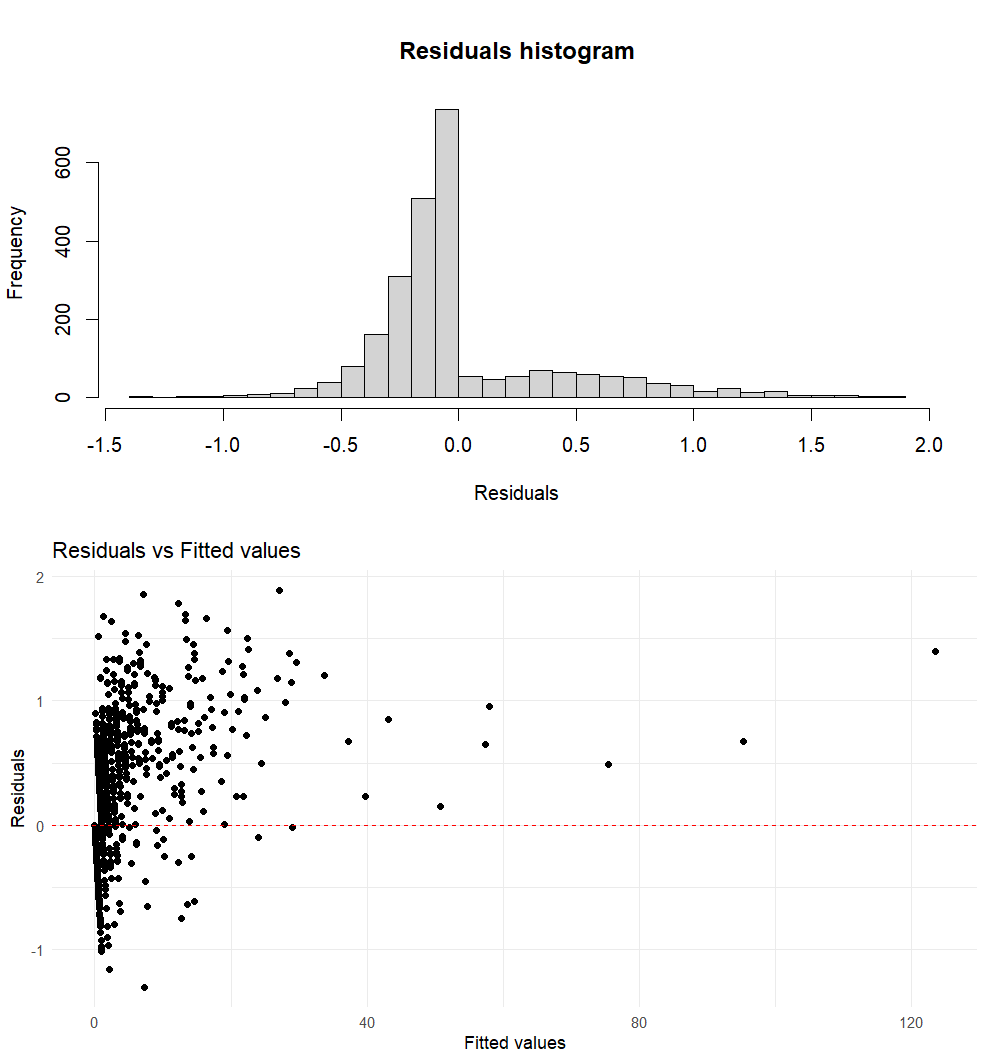

Supplement: S5 Fig — Comparison between model predictions and observed snakebite data across Mexican municipalities (2018). Total snakebite counts: model predictions (main map) and observed cases (inset). The base layer of the map showing Mexican municipalities was obtained from: https://idegeo.centrogeo.org.mx/geovisor. (INEGI, 2024). Terms of use: https://www.inegi.org.mx/contenidos/inegi/doc/terminos_info.pdf (TIF) [file pntd.0013582.s004.tif]

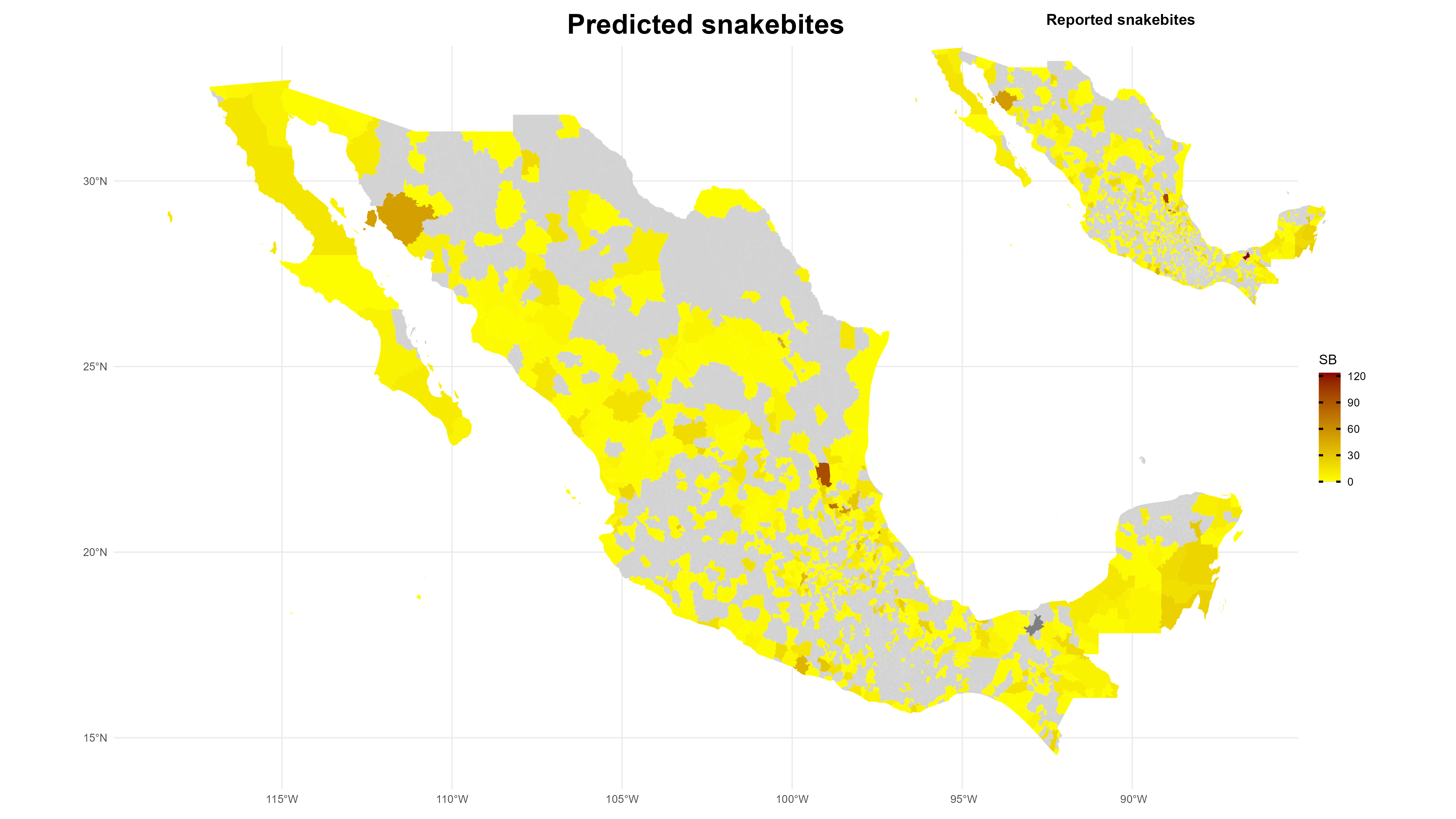

Supplement: S6 Fig — Comparison between model predictions and observed snakebite data across Mexican municipalities (2018). Population-adjusted snakebite incidence (main map) and observed cases (inset). The base layer of the map showing Mexican municipalities was obtained from: https://idegeo.centrogeo.org.mx/geovisor. (INEGI, 2024). Terms of use: https://www.inegi.org.mx/contenidos/inegi/doc/terminos_info.pdf (TIF) [file pntd.0013582.s005.tif]

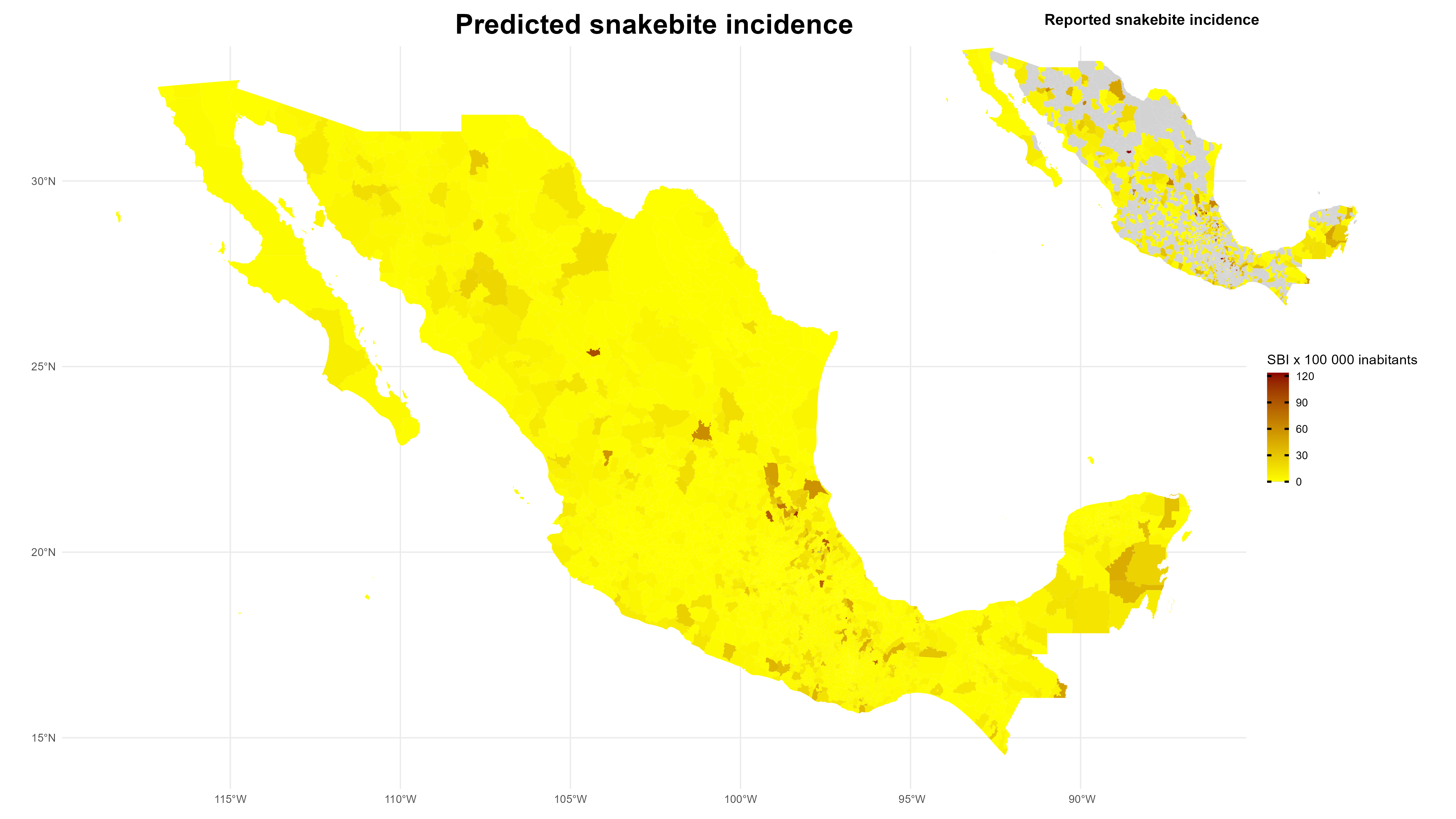

Supplement: S7 Fig — Map of municipalities with differences (red) between SB and PSB by the CAR model with a linear model between SB and PSB. The base layer of the map showing Mexican municipalities was obtained from: https://idegeo.centrogeo.org.mx/geovisor. (INEGI, 2024) Terms of use: https://creativecommons.org/licenses/by-nc/2.5/mx/. (TIF) [file pntd.0013582.s006.tif]

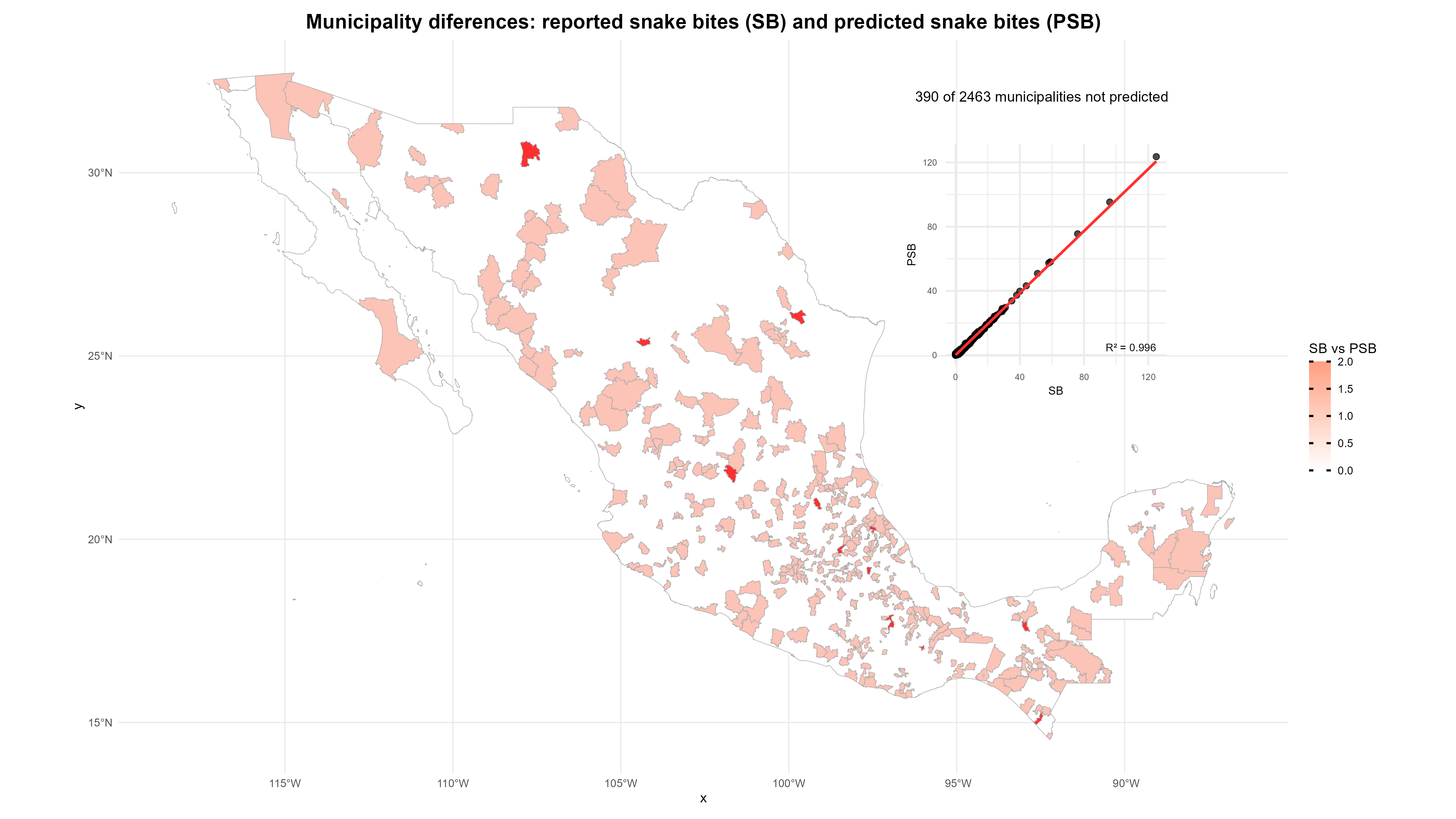

Supplement: S8 Fig — Map of municipalities with B. asper suitability different of 0 in red. The base layer of the map showing Mexican municipalities was obtained from: https://idegeo.centrogeo.org.mx/geovisor. (INEGI, 2024). Terms of use: https://www.inegi.org.mx/contenidos/inegi/doc/terminos_info.pdf (TIF) [file pntd.0013582.s007.tif]
